# Pelletized Growth in *Cordyceps militaris* Is Associated with Coordinated Cell Wall Remodeling and Stress Defense

**DOI:** 10.3390/jof12050362

**Published:** 2026-05-15

**Authors:** Na Wu, Xiaoxuan Du, Chaowei Huang, Xinru Xu, Wenke Hu, Suai Yin, Xiaoxiao Ma, Rong Shao, Kyung-Min Kim, Wei Xu

**Affiliations:** 1 School of Marine and Bioengineering, Yancheng Institute of Technology, Yancheng 224051, China; nwu@cibt.ac.cn (N.W.); 17895266026@163.com (X.D.); abcd255240@163.com (X.X.); 18215418263@163.com (W.H.); 15358660311@163.com (S.Y.); 19708233729@163.com (X.M.); sr@ycit.edu.cn (R.S.); 2 Biomedical Engineering, City University of Hong Kong, Dongguan 523000, China; 15961989720@163.com; 3 Coastal Agriculture Research Institute, Kyungpook National University, Daegu 41566, Republic of Korea; 4 Department of Applied Biosciences, Kyungpook National University, Daegu 41566, Republic of Korea

**Keywords:** *Cordyceps militaris*, exopolysaccharide, pellet formation, seed-stage morphology induction, submerged fermentation

## Abstract

Morphological control in submerged fermentation is a well-established method for enhancing bioactive metabolite production in filamentous fungi. However, the molecular mechanisms linking morphology to fermentation efficiency remain insufficiently understood. In this study, supplementing 1.5% Tween 80 (P80) at the seed culture stage of *Cordyceps militaris* consistently induced the formation of compact, uniform mycelial pellets. This morphological induction at the seed stage enhanced fermentation performance, increasing exopolysaccharide (EPS) titer by 71.1% and reducing the production cycle by 24 h. Transcriptomic analysis revealed that pelletized cultures exhibited transcriptional patterns associated with MAPK signaling related to cell wall integrity and upregulation of genes involved in cell wall remodeling. Additionally, pelletized cultures displayed a reduced oxidative burden and were associated with enhanced antioxidant capacity. These findings link morphology induction to cell wall remodeling and oxidative stress defense, offering a potentially scalable strategy for industrial polysaccharide production in medicinal fungi.

## 1. Introduction

*Cordyceps militaris* (*C. militaris*) is a well-known entomopathogenic fungus that has garnered significant attention due to its abundant bioactive metabolites, including cordycepin, cordycepic acid, adenosine, vitamins, and polysaccharides [1,2]. Among these, polysaccharides are particularly valued for their diverse pharmacological properties, such as antioxidant, immunomodulatory, antitumor, hypoglycemic, antibacterial, and anti-inflammatory activities [3,4]. Despite the growing market demand, the limited availability of wild *C. militaris* resources—restricted by specific environmental conditions—has hindered large-scale applications [5]. Consequently, submerged liquid fermentation has emerged as a preferred industrial method due to its shorter cultivation cycle, stable yields, and consistent product quality [6,7]. However, current polysaccharide yields in filamentous fungal fermentations often fall short of economic expectations, emphasizing the need for further optimization of cultivation strategies.

In submerged cultivation, filamentous fungi typically exhibit three distinct macro-morphologies: dispersed hyphae, fluffy mats, and dense pellets [8]. These morphological forms significantly affect mass transfer efficiency and dissolved oxygen levels, thereby directly influencing fermentation performance. Compact and uniform mycelial pellets are generally considered the optimal morphology for industrial bioprocessing, as they reduce broth viscosity, enhancing nutrient and oxygen diffusion within the bioreactor [9,10]. Several strategies have been developed to modulate fungal morphology, including adjustments to nitrogen-to-carbon ratios and the addition of inorganic particles or specific surfactants [11,12,13,14]. Notably, Tween 80 (P80), a non-ionic surfactant, has shown promise in inducing pellet formation in various species. For instance, Liu et al. demonstrated that combining Tween 80 (P80) with pH 6.0 effectively induced pelletization in *Cordyceps sinensis*, leading to a doubling of exopolysaccharide (EPS) yield [15]. However, the use of P80 to regulate *C. militaris* morphology remains underexplored, and the molecular mechanisms driving this transition, along with the relationship between morphology and metabolic efficiency, are not yet fully understood.

The morphological evolution of filamentous fungi is fundamentally a result of dynamic cell wall remodeling [16]. This process is regulated by a complex signaling network, with the mitogen-activated protein kinase (MAPK) cascade playing a pivotal role in maintaining cell wall integrity (CWI) and responding to external environmental stimuli [17]. Key MAPK pathway genes have been shown to regulate growth and secondary metabolism in various fungal species, including *Coprinopsis cinerea*, *Neurospora crassa*, and *Pleurotus ostreatus* [18,19,20]. Additionally, industrial fermentation subjects cells to oxidative stress due to factors such as shear forces, nutrient depletion, and metabolite accumulation, which generate reactive oxygen species (ROS) and cause subsequent cellular damage [21]. Thus, enhancing the endogenous antioxidant defense system and biosynthesis of protective osmolytes is essential for maintaining physiological robustness and metabolic flux during high-density fermentation.

This study developed a straightforward morphological engineering strategy by adding P80 during the seed culture stage of *C. militaris* to induce uniform pellet formation. To differentiate the effects of morphology from those of the surfactant itself during fermentation, a wash-and-transfer approach was employed. Seed cultures pretreated with P80 were washed and inoculated into P80-free fermentation media, allowing us to assess whether morphology induction at the seed stage alone could improve EPS productivity. By integrating fermentation phenotyping with transcriptomic profiling and biochemical assays, this study evaluated the impact of P80-induced pelletization on EPS synthesis and investigated the related molecular and physiological features. The primary objectives were to determine whether seed-stage P80 treatment consistently promotes a pellet morphology that enhances fermentation performance and to characterize the transcriptional and biochemical correlates of pelletized growth, including cell wall remodeling and oxidative stress responses. These findings aim to provide practical insights for the rational design of fungal cell factories through morphological control.

## 2. Materials and Methods

### 2.1. Strains, Media and Growth Conditions

Three types of media were prepared for different purposes. Potato dextrose agar (PDA) medium was used for strain maintenance. It was prepared by peeling and chopping 200 g of fresh potatoes, boiling for approximately 30 min, filtering, and adjusting the volume to 1 L with water to obtain a 20% (*w*/*v*) potato extract. Subsequently, 20 g glucose and 15 g agar were added, and the medium was sterilized at 115 °C for 30 min. Seed culture medium was used for inoculum preparation and consisted of 20 g/L glucose, 10 g/L peptone, 3 g/L KH_2_PO_4_, and 1.5 g/L MgSO_4_, dispensed as 100 mL aliquots in 250 mL flasks and sterilized at 115 °C for 30 min. Fermentation medium was used for exopolysaccharide (EPS) production and contained 40 g/L glucose, 10 g/L peptone, 0.5 g/L KH_2_PO_4_, 0.5 g/L MgSO_4_, and 0.5 g/L CaCO_3_, similarly sterilized at 115 °C for 30 min.

*C. militaris* 01 (obtained from the China Center of Industrial Culture Collection, CCICC) was inoculated onto PDA medium and incubated at 25 °C for 5 d [22]. The spores were collected using sterile water and counted using a hemocytometer to determine the total number of spores (viability was not assessed). Before inoculation, the suspension was adjusted to a total spore concentration of 1 × 10^7^ spores/mL. Viability was not assessed; however, this was consistent across all experimental groups and did not interfere with the comparative results. The spores were then inoculated into the seed culture medium, followed by incubation at 25 °C and 180 rpm for 5 d.

### 2.2. Surface Active Agent Treatments

**Figure 1:**
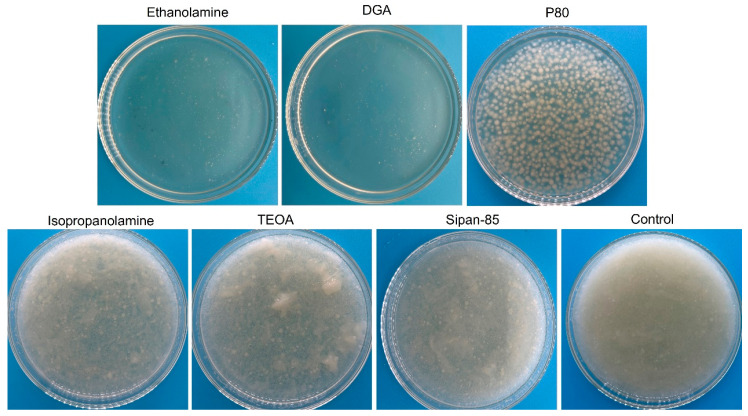
Representative images of *C. militaris* after seed culture medium was added with different surfactants (1.5% *v*/*v*).

For seed culture experiments, 1% (*v*/*v*) of various surfactants, including ethanolamine, diethylene glycol amine (DGA), triethanolamine (TEOA), P80 (tested at concentrations of 0.5%, 1.0%, 1.5%, and 2.0%), sorbitan trioleate (Sipan-85), and isopropanolamine, were individually added to 100 mL seed medium. A no-surfactant control was also included. After thorough mixing, the media were inoculated with spores and incubated at 25 °C and 180 rpm for 5 d. As shown in Figure 1 and described in the Results, only P80 among these surfactants reproducibly induced compact pellet formation without apparent cytotoxicity (ethanolamine and DGA severely inhibited growth; isopropanolamine, TEOA, and Sipan-85 showed negligible morphological effects). Therefore, P80 was selected for further concentration optimization, and the data for the four P80 concentrations (0.5%, 1.0%, 1.5%, and 2.0%) were used for subsequent analyses. Seed cultures were washed three times with sterile water by centrifugation (3354 *g*, 10 min), with complete supernatant removal after each wash. We did not perform analytical measurements (e.g., HPLC) to confirm residual P80 absence; trace carryover cannot be formally excluded. The three-step washing was standard practice [15].

### 2.3. Morphological Observation

After 5 d of seed culture, fungal suspensions were transferred to sterile disposable Petri dishes for macroscopic observation and photographic documentation of morphology under different treatments using a light microscope (CX23, Olympus, Tokyo, Japan). For ultrastructural examination, samples from control (0% P80) and 1.5% P80-treated seed cultures were collected after 5 days of growth and processed for field-emission scanning electron microscopy (FE-SEM; S-4800, Hitachi, Tokyo, Japan). Briefly, samples were fixed in 2.5% glutaraldehyde and rinsed three times with phosphate-buffered saline (PBS). Subsequently, specimens were dehydrated through a graded ethanol series (30%, 50%, 70%, 80%, 90%, 95%, and 100%), with each step lasting 15 min. The dehydrated samples were then freeze-dried prior to SEM imaging to examine hyphal ultrastructure, including hyphal arrangement, surface smoothness, and aggregation patterns.

### 2.4. Fermentation Parameter Measurement

Following established protocols [23], mycelia from eggplant flask cultures were washed with sterile water and inoculated into 100 mL seed medium at 25 °C, 180 rpm for 5 d to generate seed culture. For P80-pretreated groups, washed seed cultures were used as inoculum. Then, 1% (*v*/*v*) seed inoculum was transferred to 100 mL fermentation medium for 7 d under the same conditions. Mycelia from seed and fermentation stages were collected by filtration through five layers of sterile gauze, washed thrice with deionized water, and dried at 50 °C to constant weight for biomass determination. Fermentation broth was centrifuged at 4 °C, 3354 *g* for 10 min to obtain supernatant for residual glucose and EPS analysis. Glucose concentration was measured using an SBA-40C biosensor analyzer (Jinan Yanhe Biotechnology, Jinan, China) after 100-fold dilution. Crude EPS was precipitated by adding four volumes of cold absolute ethanol to the fermentation broth, followed by incubating at 4 °C for 12 h, followed by centrifugation at 3354 *g* for 15 min. The precipitate was dissolved in 1 M NaOH and incubated at 60 °C for 1 h. EPS content in the supernatant was quantified by the phenol-sulfuric acid method as previously described [24].

### 2.5. Biochemical Analysis

Samples from control and experimental groups were collected on 1, 3, and 7 d of fermentation, with three independent biological replicates (separate flasks started from independent inoculum). Data were presented as mean ± SD of these three replicates. Mycelia were thoroughly washed with deionized water to remove residual CaCO_3_, accurately weighed, and processed for analysis. Samples were prepared by ice-cold grinding at a ratio of 0.1 g tissue per 1 mL extraction buffer, followed by ultrasonic cell disruption (300 W, 3 s on/7 s off cycles, total 3 min). After centrifugation at 8000 *g* and 4 °C for 10 min, supernatants were collected and stored on ice for metabolite assays using commercial kits from Beijing Solarbio Technology Co., Ltd. (Beijing, China), following manufacturer instructions.

### 2.6. Transcriptomic Analysis

At 3 d of seed culture, mycelial samples from untreated (fluffy morphology) and 1.5% P80-treated (pellet morphology) groups were collected from three independent biological replicates per condition. The 1.5% P80 concentration was selected for transcriptomic analysis because, among the concentrations tested (0.5%, 1.0%, 1.5%, and 2.0%), it yielded the most compact and uniform pellets with the smallest average diameter and the highest biomass accumulation during seed culture (as assessed by microscopic examination and dry cell weight measurement). Each replicate was processed individually for RNA extraction and library preparation, and sequenced separately (no pooling), centrifuged to remove supernatant, and immediately frozen at −80 °C. Total RNA was extracted using standard protocols. RNA quantity and purity were assessed by Nanodrop 2000 (Thermo Fisher Scientific, Waltham, MA, USA), and integrity was checked by agarose gel electrophoresis and an Agilent 2100 Bioanalyzer (Agilent Technologies, Santa Clara, CA, USA), with quality criteria set as total RNA ≥ 1 μg, concentration ≥ 35 ng/μL, OD260/280 ≥ 1.8, and OD260/230 ≥ 1.0. mRNA with poly-A tails was enriched using oligo(dT) beads and fragmented randomly. First-strand cDNA synthesis was primed with random hexamers, followed by second-strand synthesis. After end repair and A-tailing, adaptors were ligated to construct sequencing libraries. Sequencing was performed on the Illumina NovaSeq 6000 platform (Illumina, San Diego, CA, USA). Transcriptomic data processing and bioinformatics analyses were conducted by Shanghai Meiji Biomedical Technology Co., Ltd. (Shanghai, China).

### 2.7. RT-qPCR Analysis

Total RNA was extracted from *C. militaris* samples using the Vazyme RNA extraction kit and reverse transcribed into cDNA with the HiScript III RT kit (Vazyme, Nanjing, China). Quantitative PCR was performed using SYBR Green chemistry on the Applied Biosystems QuantStudio 3 system (Thermo Fisher Scientific, Waltham, MA, USA) [25]. Gene expression levels were calculated by the 2^−ΔΔCT^ method, normalized against β-actin as the internal control. Primer sequences are provided in Table S1 (Supplementary Files). All assays included three biological replicates.

### 2.8. Statistical Analysis

All experiments were performed with three independent biological replicates unless otherwise specified. Data were presented as mean ± standard deviation (SD). For the P80 concentration optimization experiment (0.5%, 1.0%, 1.5%, and 2.0% *v*/*v*, plus no-surfactant control), a completely randomized design was used. Differences in pellet diameter and dry cell weight across concentrations were analyzed by one-way analysis of variance (ANOVA) followed by Tukey’s post hoc test for multiple comparisons. For comparisons between two groups (e.g., control vs. 1.5% P80 in fermentation performance), a two-tailed Student’s *t*-test was used. A *p*-value < 0.05 was considered statistically significant. All statistical analyses were performed using GraphPad Prism 8.0 (GraphPad Software, San Diego, CA, USA) or SPSS 22.0 (IBM, Armonk, NY, USA). For transcriptomic analysis, differentially expressed genes were identified using DESeq2 with an adjusted *p*-value (FDR) < 0.05 and |log_2_ fold change| ≥ 1 as thresholds.

## 3. Results

### 3.1. Effect of Surfactants on the Morphology of C. militaris

To optimize the morphology of *C. militaris* at the seed culture stage, six surfactants were screened for their ability to modulate initial mycelial architecture (Figure 1). The surfactant-free control group exhibited fluffy, filamentous mycelia with loose aggregation, representing the typical dispersed morphology under the tested conditions. In contrast, isopropanolamine, TEOA, and Sipan-85 showed minimal effects on mycelial organization, while ethanolamine and DGA severely inhibited mycelial growth. Notably, only Tween 80 (P80) consistently induced a marked morphological transition, converting the dispersed and irregular mycelial aggregates observed in the control into well-defined, spherical pellets, making P80 the most effective surfactant among those tested.

**Figure 2:**
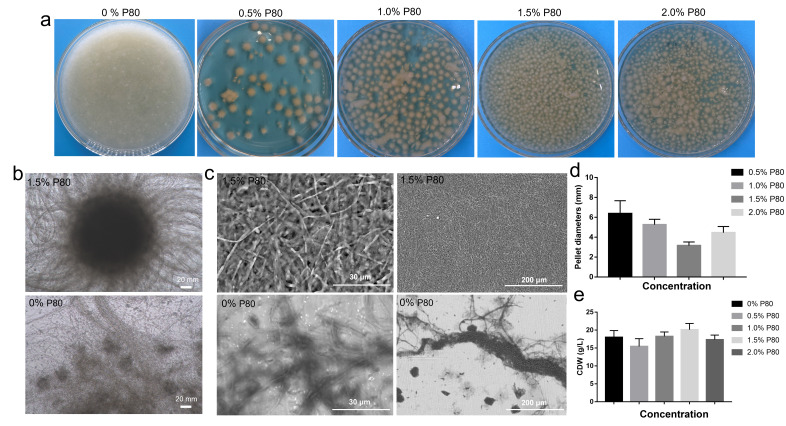
Optimization of P80 concentration in *C. militaris* seed culture medium. (**a**) Morphology of *C. militaris* spores under different P80 concentrations. (**b**) Images comparing spores cultured without P80 and with 1.5% P80. (**c**) SEM images of *C. militaris* without P80 and with 1.5% P80. (**d**) Pellet size distribution as a function of P80 concentration. (**e**) Effect of varying P80 concentrations on dry cell weight. Data represented mean ± standard deviation from three independent experiments. Statistical significance was determined by one-way ANOVA with Tukey’s post hoc test (n = 3).

Given that P80 uniquely altered the initial growth form, its concentration was further optimized within the range of 0.5–2.0% (*v*/*v*) using a completely randomized design with three independent biological replicates per concentration. Visual and microscopic observations (Figure 2a,b) revealed that pellet morphology was highly sensitive to P80 concentration. The 1.5% P80 concentration produced the most compact and uniform pellets, while both lower (0.5%) and higher (2.0%) concentrations resulted in larger, more heterogeneous aggregates. To assess whether pelletized growth was associated with structural changes in the hyphal surface, scanning electron microscopy (SEM) was performed on the control and 1.5% P80-induced pelletized cultures (Figure 2c). Compared to the dispersed control, pelletized mycelia exhibited a more compact and ordered hyphal arrangement, with noticeable alterations in surface architecture. These observations suggest that the ultrastructural differences observed were linked to altered hyphal surface organization in pelletized cultures, potentially reflecting cell wall restructuring during pellet development and maintenance.

Quantitative analysis of pellet size distribution (Figure 2d) confirmed that the smallest average pellet diameter (3.15 ± 0.37 mm) was achieved at 1.5% P80, significantly smaller than the diameters at 1.0% (5.62 ± 0.54 mm, *p* < 0.01) and 2.0% (4.46 ± 0.62 mm, *p* < 0.05), as determined by one-way ANOVA with Tukey’s post hoc test. Correspondingly, biomass accumulation reached a maximum of 20.11 ± 1.16 g/L in the 1.5% P80 group (Figure 2e), representing a significant increase compared to the surfactant-free control (18.02 ± 1.87 g/L, *p* < 0.05). In contrast, biomass declined at 2.0% P80. These results demonstrate that P80 acts as an effective morphology induction agent during the seed culture stage, with 1.5% identified as the optimal concentration for generating small, uniform pellets while maintaining robust biomass accumulation. This optimized seed morphology provided a controlled and reproducible starting point for subsequent fermentation, independent of any downstream metabolic effects.

### 3.2. Effect of P80-Induced Morphology on the Fermentation Performance of C. militaris

**Figure 3:**
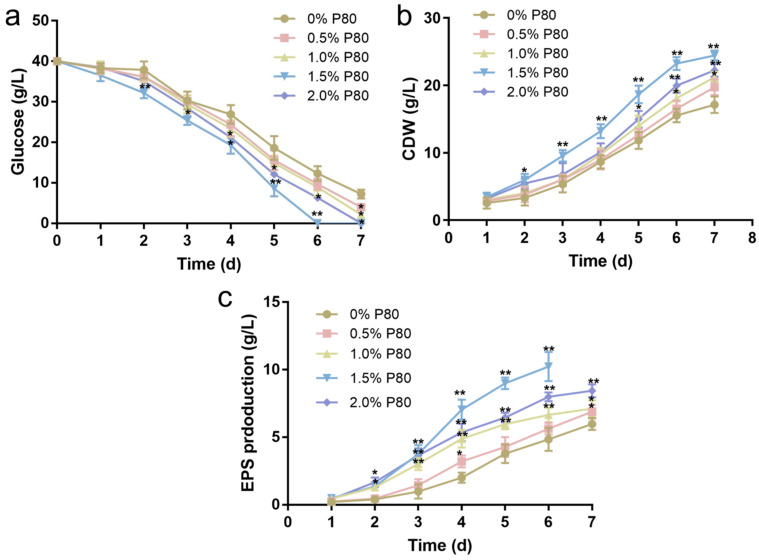
Effects of different P80 concentrations on *C. militaris* fermentation performance. (**a**) Glucose concentration. (**b**) Cell dry weight. (**c**) EPS production. Data are presented as mean ± SD (n = 3). Statistical significance was determined by Student’s *t*-test. * *p* < 0.05, ** *p* < 0.01.

To explicitly distinguish morphology-mediated effects from any direct contribution of P80 during fermentation, seed cultures pretreated with P80 were thoroughly washed to remove residual surfactant and subsequently inoculated into fresh fermentation media without P80. Although the seed cultures were washed three times, trace residual P80 cannot be formally excluded in the absence of analytical confirmation (e.g., HPLC-MS). Future studies should therefore include quantitative residual surfactant analysis (e.g., HPLC-MS) to definitively exclude carryover effects. Under these conditions, the fermentation performance of the P80-pretreated cultures remained markedly distinct from that of the untreated control. As shown in Figure 3a, cultures derived from seeds pretreated with 1.5% P80 exhibited substantially accelerated glucose consumption, with complete substrate depletion achieved by day 6, whereas control cultures retained 12.30 ± 1.80 g/L of residual glucose at the same time point. This difference in substrate utilization was accompanied by a pronounced divergence in biomass accumulation. The 1.5% P80 group reached a maximum dry cell weight of 24.45 ± 0.73 g/L, representing a 42.7% increase compared to the control group (17.13 ± 1.23 g/L) (Figure 3b). Notably, this growth advantage emerged during fermentation, despite comparable or lower biomass levels in the seed cultures.

As shown in Figure 3c, the 1.5% P80 group achieved the highest maximum EPS titer (10.23 ± 1.07 g/L by day 6), nearly doubling the control value (5.98 ± 0.44 g/L at day 7). The 0.5% and 1.0% P80 groups showed intermediate EPS yields of 6.89 ± 0.52 g/L and 7.12 ± 0.61 g/L, respectively, while the 2.0% group produced 8.45 ± 0.48 g/L, consistent with its less uniform pellet morphology and reduced biomass. One-way ANOVA with Tukey’s post hoc test confirmed that the 1.5% P80 group yielded significantly higher EPS than all other groups (*p* < 0.01 vs. control; *p* < 0.05 vs. 0.5%, 1.0%, and 2.0%). Importantly, the improvement for the 1.5% P80 group was reflected not only in a higher final titer but also in an earlier peak production (day 6 vs. day 7 for control).

### 3.3. Transcriptomic Analysis of the Mechanism Underlying Pellet Formation in C. militaris

**Figure 4:**
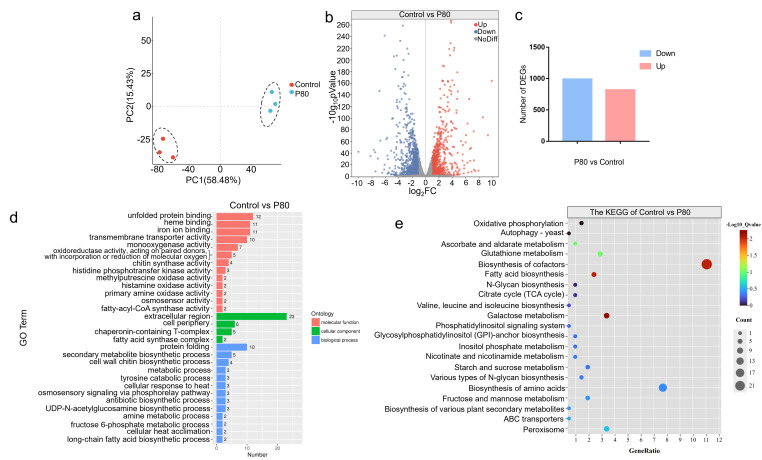
Transcriptome analysis and annotation of differentially expressed genes in 1.5% P80 vs. 0% P80 seed cultures. (**a**) PCA plot showing separation between 1.5% P80 and control groups. (**b**) Volcano plot of differentially expressed genes. (**c**) Number of upregulated and downregulated genes. (**d**) Gene Ontology (GO) annotation of DEGs. (**e**) KEGG pathway enrichment of DEGs.

To characterize the transcriptional features associated with pellet formation, comparative transcriptomic analysis was performed between seed cultures exhibiting dispersed mycelial morphology (0% P80) and those forming uniform pellets following 1.5% P80 pretreatment. Principal component analysis (PCA) revealed clear separation between the two groups (Figure 4a), indicating that pelletized and dispersed cultures occupy distinct transcriptional states at the seed stage. In total, 1813 differentially expressed genes (DEGs) were identified, comprising 992 upregulated and 821 downregulated genes in the pelletized group relative to the control (Figure 4b,c). Gene Ontology (GO) annotation and Kyoto Encyclopedia of Genes and Genomes (KEGG) pathway enrichment analyses (Figure 4d,e) revealed that these DEGs were primarily associated with metabolic processes, membrane components, and catalytic activities. Notably, pathways related to oxidative phosphorylation, glutathione metabolism, and lipid metabolism were significantly enriched.

**Figure 5:**
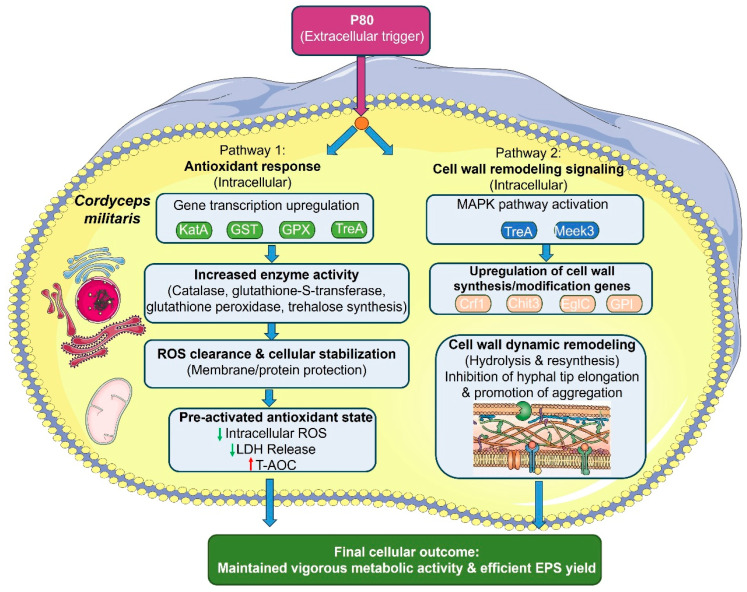
Mechanism of cell wall remodeling signaling in P80-induced pellet formation of *C. militaris*. Expression changes for Ste50 and Mekk3 were not statistically significant (FDR > 0.05) and are shown for directional information only.

Among the enriched functional categories, genes involved in cellular stress defense were notably overrepresented. Several antioxidant-related genes showed increased expression in pelletized cultures, including catalase (KatA, CCM_02506, 1.46-fold), glutathione S-transferase (GST, CCM_02549, 4.02-fold), and glutathione peroxidase (GPX, CCM_03086, 1.09-fold) (Figure 5; Table S2). In addition, the trehalase precursor gene TreA (CCM_08782) was modestly upregulated (1.12-fold). Transcriptomic profiling also revealed differential expression of genes involved in the MAPK CWI signaling module. The adaptor protein Ste50 (CCM_09426) and the upstream kinase Mekk3 (CCM_01278) exhibited directional changes (log_2_FC = 0.21 and 0.11, corresponding to 1.16- and 1.08-fold, respectively) in pelletized cultures. It should be noted that these changes did not meet the pre-specified thresholds for differential expression (|log_2_FC| ≥ 1, FDR < 0.05) and are therefore presented only as non-significant trends. These trends suggest potential involvement of CWI-related signaling components during pellet development and maintenance, though further validation is needed. In parallel, genes encoding enzymes directly involved in cell wall remodeling were strongly upregulated. Notably, the β-glucanase Crf1 (CCM_02110), chitinase Chit3 (CCM_07121), and the GPI-anchored endoglucanase EglC (CCM_04122) exhibited significant increases in expression (258%, 98.6%, and 35.2%, respectively) in pelletized cultures compared to the dispersed control (Figure 5; Table S2).

### 3.4. Validation of RNA Seq by qRT-PCR

**Figure 6:**
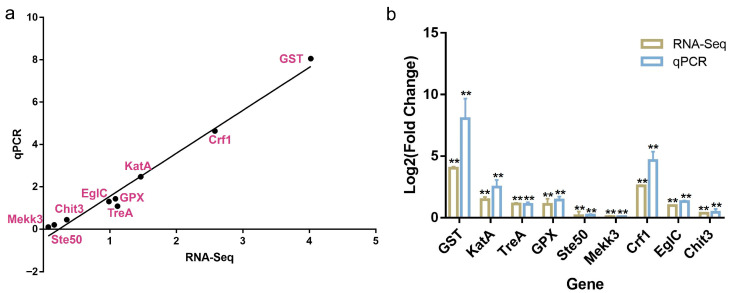
Validation of transcriptome data. (**a**) Linear correlation analysis between qPCR and RNA-seq results. (**b**) qPCR validation of RNA-seq data, with values presented as mean ± standard deviation. Data are presented as mean ± SD (n = 3). Statistical significance was determined by Student’s *t*-test. ** *p* < 0.01.

To assess the reliability and reproducibility of the transcriptomic analysis, nine representative genes associated with stress response and cell wall remodeling were selected for validation via qRT-PCR. As shown in Figure 6a, the expression trends observed by qRT-PCR closely matched those from RNA-seq analysis, demonstrating strong concordance between the two methods. Linear regression analysis revealed a significant positive correlation between qRT-PCR and RNA-seq data (r^2^ = 0.9793).

Specifically, compared to the dispersed-morphology control group (0% P80), genes involved in oxidative stress defense, including GST, GPX, TreA, and KatA, exhibited significantly higher expression levels in pelletized cultures derived from 1.5% P80-pretreated seeds (Figure 6b). Similarly, genes associated with cell wall remodeling and the MAPK CWI-related module, including Mekk3, Crf1, Ste50, Chit3, and EglC, were also upregulated in these cultures.

### 3.5. Biochemical Analysis

**Figure 7:**
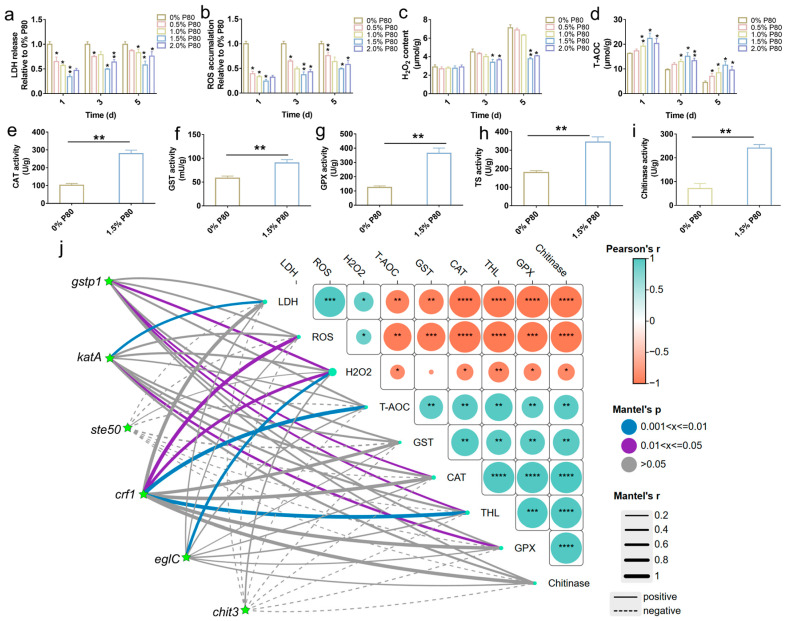
Biochemical analysis of the stress defense system in *C. militaris*. (**a**) LDH assay. (**b**) ROS assay. (**c**) H_2_O_2_ assay. (**d**) T-AOC assay. (**e**) CAT assay. (**f**) GST assay. (**g**) GPX assay. (**h**) TS assay. (**i**) Chitinase assay. (**j**) Mantel test showing correlations between physiological parameters (LDH, ROS, T-AOC, CAT, GST, GPX, TS, chitinase) and expression levels of corresponding genes (KatA, Gstp1, Crf1, EgIC, Chit3). Line color represents Mantel’s r statistic (Blue indicates *p* ≤ 0.01, purple indicates *p* ≤ 0.05, and gray indicates no significance); line width represents significance (thicker lines indicate *p* < 0.05, thickest lines indicate *p* < 0.001). Matrix cells on the right show pairwise correlations among physiological parameters (color intensity indicates Pearson’s r). Data are presented as mean ± SD (n = 3). Statistical significance was determined by Student’s *t*-test. * *p* < 0.05, ** *p* < 0.01, *** *p* < 0.001, **** *p* < 0.0001.

To characterize the physiological state associated with pelletized growth, key biochemical markers related to membrane integrity, oxidative stress, and cell wall remodeling were monitored during cultivation. Lactate dehydrogenase (LDH) activity was significantly lower in cultures derived from 1.5% P80-pretreated seeds compared to the dispersed-morphology control (Figure 7a). Pelletized cultures consistently exhibited lower ROS accumulation than the control throughout cultivation (Figure 7b). Concurrently, the total antioxidant capacity (T-AOC) in the pelletized group reached a maximum of 11.56 ± 1.61 μmol/g by day 6, representing a 2.5-fold increase compared to the dispersed control (4.56 ± 0.57 μmol/g) (Figure 7c).

To further assess whether these physiological differences corresponded to changes in enzymatic activities, several key antioxidant enzymes were analyzed. Catalase (CAT) activity in pelletized cultures reached 279.31 ± 19.74 U/g protein, a 174.83% increase relative to the control (Figure 7e). Similarly, GST and GPX exhibited increases of 54.7% and 191.8%, respectively (Figure 7f,g). Additionally, trehalose synthase (TS) activity was elevated by 91.9% in pelletized cultures (Figure 7h).

In addition to stress-related parameters, biochemical markers associated with cell wall dynamics were also examined. Chitinase activity, which plays a pivotal role in hyphal restructuring and morphological adaptation [26], increased by approximately 240% in pelletized cultures relative to the control (Figure 7i). The elevated chitinase activity observed in pelletized cultures suggests enhanced activity of enzymes involved in cell wall modification. These findings, coupled with SEM-observed ultrastructural differences and the upregulation of cell wall-related genes, support the idea that pelletized growth is linked to active cell wall remodeling. To evaluate the coherence between physiological measurements and molecular responses, a Mantel test was performed to integrate biochemical indicators with corresponding gene expression data (Figure 7j). Significant correlations (*p* < 0.001) were observed between physiological parameters (LDH, ROS, T-AOC, CAT, GST, GPX, TS, and chitinase activity) and the expression of their associated genes (e.g., KatA, Gstp1, Crf1, EglC, and Chit3).

## 4. Discussion

The development of robust microbial cell factories to convert renewable biomass into high-value biochemicals is a cornerstone of the global transition toward a sustainable bio-economy [27]. However, the industrial-scale cultivation of filamentous fungi, such as *C. militaris*, is often hindered by intrinsic bioprocess bottlenecks, including oxidative stress, product-induced inhibition, and excessive mycelial entanglement. These factors collectively increase broth viscosity and cause severe mass transfer limitations, ultimately compromising cellular growth and metabolic productivity [28]. To address these challenges, morphological engineering through surfactant supplementation has emerged as an effective bioprocess intervention. This strategy promotes the transition from dispersed, tangled hyphae to uniform, dense pellets, thereby optimizing the rheological properties of the fermentation system and significantly enhancing oxygen and nutrient diffusion efficiency [15]. While empirical evidence has demonstrated the efficacy of various surfactants in modulating fungal morphology and alleviating fermentation-associated stresses, the underlying molecular mechanisms and biochemical regulatory networks driving these transitions remain largely unexplored. Consequently, this study investigated whether a simple seed-stage surfactant intervention could induce a stable pelletized growth state in *C. militaris* and whether this morphology, once established and surfactant removal completed, would lead to sustained improvements in fermentation performance.

Our results demonstrated that 1.5% P80 in seed culture reliably induced uniform pellets, which maintained their performance advantage in P80-free fermentation, establishing a direct link between seed morphology induction and enhanced EPS productivity. Importantly, the fermentation advantage persisted even after the P80-pretreated seeds were washed and transferred into P80-free fermentation medium (Figure 3). Since P80 was absent from the fermentation medium, these improvements can be attributed to the retained effects of seed-stage morphology induction, rather than any direct stimulatory role of the surfactant during EPS biosynthesis. This structural optimization resulted in a significant 71.1% increase in EPS yield, from 5.98 ± 0.44 g/L in the control to 10.23 ± 1.07 g/L (Figure 3c), reinforcing the paradigm that pelletized morphology is often more effective for high-titer secondary metabolite biosynthesis in filamentous fungi [29]. However, a distinct threshold effect was observed; P80 concentrations above 1.5% led to heterogeneous macro-aggregates and inhibited biomass growth. These findings indicate an optimal concentration window for effective morphology induction, rather than a simple linear dose–response relationship. In contrast, biomass declined at 2.0% P80, suggesting that excessive surfactant exposure may disrupt normal hyphal development, despite promoting aggregation. Overall, these results show that P80 serves as an effective morphology-inducing agent during the seed culture stage, with 1.5% identified as the optimal concentration for generating small, uniform pellets while maintaining robust biomass accumulation. This optimized seed morphology provided a controlled and reproducible starting state for subsequent fermentation, independent of any downstream metabolic effects. This enhancement implies that optimized pellet morphology is compatible with, rather than detrimental to, vegetative growth during seed culture, consistent with previous reports that appropriately sized pellets can alleviate mass transfer limitations and support biomass formation [30,31]. The accelerated glucose consumption and biomass accumulation in the 1.5% P80 group further emphasized the bioprocess advantages of pelletization. By optimizing the surface-to-volume ratio and reducing broth viscosity, these well-defined pellets likely alleviated mass transfer resistance and improved dissolved oxygen availability. In contrast to the control cultures, which maintained a dispersed and filamentous morphology, the P80-pretreated cultures preserved uniform and spherical pellets throughout the fermentation process—a feature typically associated with reduced broth viscosity and improved process stability. While no direct rheological measurements were conducted at this stage, the observed enhancements in glucose utilization and biomass formation align with improved bioprocess performance in pelletized filamentous fungi. Notably, the earlier achievement of peak EPS titer (day 6 vs. day 7) suggests a genuine increase in productivity, rather than simply an extension of cultivation time, consistent with successful morphology control strategies in other high-performance microbial systems [30].

The integrated transcriptomic and biochemical analyses revealed that pelletized cultures were associated with biochemical features of antioxidant defense in *C. militaris*. In industrial submerged fermentation, environmental stressors such as hydrodynamic shear, nutrient depletion, and the accumulation of inhibitory metabolites inevitably trigger an increase in intracellular ROS, leading to oxidative damage that compromises cellular growth and metabolic efficiency [32,33,34]. Consistent with a lower damage state, pelletized cultures exhibited reduced LDH activity and lower ROS accumulation, along with elevated T-AOC (Figure 7). LDH is a commonly used indicator of cytoplasmic leakage and membrane damage [35]. The reduction in LDH activity indicates improved membrane integrity in pelletized cultures, suggesting a more structurally stable growth form under the tested conditions. These results indicate that pelletized cultures were associated with a reduced oxidative burden and enhanced antioxidant capacity, particularly in relation to oxidative stress-related gene expression and enzyme activities, as evidenced by lower ROS and H_2_O_2_ levels and elevated activities of CAT, GST, and GPX [36]. Additionally, the compact pellet morphology likely reduced exposure to hydrodynamic shear stress, although shear was not directly measured in this study. This physiological resilience was further supported by the upregulation of key antioxidant genes (KatA, GST, and GPX) and their corresponding enzymatic activities [37,38]. Notably, while KatA (1.46-fold) and GPX (1.09-fold) showed modest transcriptional upregulation, their corresponding enzymatic activities increased dramatically (CAT by 174.8%, GPX by 191.8%). GST exhibited a substantial 4.02-fold transcriptional increase, which aligned with a 54.7% increase in enzymatic activity. These observations suggest that post-transcriptional regulation or direct enzyme activation may amplify functional outcomes beyond what is predicted by transcript levels alone. Factors such as protein stability, allosteric activation, or substrate availability could contribute to these discrepancies. Future studies focusing on protein abundance and enzyme kinetics would provide further insights into the mechanisms underlying these observations. GST and GPX, both of which are involved in the detoxification of electrophilic compounds and ROS [39], were upregulated in pelletized cultures. Additionally, the upregulation of genes associated with trehalose biosynthesis, particularly TreA, likely contributed to the stabilization of proteins and membrane structures under fluctuating fermentation conditions [40]. The modest upregulation of TreA suggests an enhanced turnover or regulation of protective osmolytes such as trehalose and glutathione, which are commonly involved in stress adaptation during high-density or aggregated growth. Furthermore, TS activity was elevated in pelletized cultures, indicating an enhanced capacity for the accumulation or turnover of trehalose, a key osmolyte linked to stress adaptation [41,42]. These findings support the notion that enhanced stress tolerance accompanies pelletized growth, potentially sustaining metabolic robustness during high-density cultivation. However, this does not necessarily imply that antioxidant activation was the sole driver of EPS biosynthesis. The coordinated increase in antioxidant enzymes (KatA, GST, GPX) and reduced ROS levels in pelletized cultures may reflect the activation of a protective stress-response pathway. Nonetheless, pellet morphology might also improve mass transfer, reduce shear stress, and enhance nutrient utilization [9,10], which could lower baseline physiological stress. In this context, reduced ROS and LDH activity might indicate a less stressful microenvironment, with elevated antioxidant capacity serving as a correlate of improved cellular fitness rather than a direct defense mechanism. Our current data did not distinguish between these possibilities. Therefore, throughout the manuscript, we have interpreted these findings as correlations rather than proven causal relationships, and we acknowledge that the reduced oxidative burden in pelletized cultures may partly result from a less stressful physical microenvironment. To resolve this, future studies should include targeted analyses such as early-stage measurements of ROS and oxidative damage markers, controlled shear-sensitivity assays, and comparative metabolic flux analysis. Given these complexities, the observed phenotype was interpreted as a correlative feature of pelletized growth, which could result from active defense mechanisms, reduced baseline stress, or a combination of both. This interpretation aligns with bioprocess intensification concepts, where adaptive stress-management capacity supports productive states under process constraints [43,44].

The transcriptomic analysis revealed correlations between pellet formation and maintenance and the expression of genes involved in cell wall remodeling pathways. Fungal morphology is fundamentally a phenotypic manifestation of dynamic cell wall plasticity [45], with morphological differentiation closely linked to the continuous remodeling of cell wall architecture [46]. Our results showed that P80 treatment was associated with directional changes in the expression levels of components of the MAPK signaling cascade, particularly the adaptor protein Ste50 and the kinase Mekk3. These changes did not meet the statistical significance thresholds (|log_2_FC| ≥ 1, FDR < 0.05) and should be interpreted only as non-significant trends. Rather than indicating pathway activation, these directional trends suggest a possible, but unconfirmed, involvement of CWI-related signaling components during pellet development and maintenance, which warrants further investigation with targeted approaches. Ste50 functions as a global regulator in various filamentous fungi, orchestrating hyphal development, differentiation, and secondary metabolism through downstream MAPK modules [47,48,49]. In this system, the modest transcriptional changes in Ste50 and Mekk3 align more with the engagement of a CWI-related response during pelletization than with conclusive evidence of pathway activation. The transition from dispersed filaments to compact pellets requires extensive re-engineering of the cell wall [50]. In addition to cell wall enzymes, recent structural studies in ascomycetes have identified BAR domain-containing proteins (such as Ssp1 in *S. cerevisiae*) that regulate membrane curvature and plasma membrane organization, which may contribute to the ultrastructural surface alterations observed in pelletized hyphae [51]. Microscopic observations confirmed that P80-treated mycelia exhibited reduced hyphal tip extension, indicating a shift from longitudinal growth to lateral aggregation, a process that demands continuous stretching and reconstruction of the cell wall matrix. This remodeling was driven by rapid enzymatic hydrolysis and resynthesis of structural polymers, particularly chitin and β-glucan [52]. Consistent with this, our transcriptomic data showed increased expression of genes encoding key cell wall-modifying enzymes, including β-glucanase (Crf1), chitinase (Chit3), GPI-anchored proteins, and endoglucanase (EglC). Since fungal cell walls are constantly remodeled during growth and morphogenesis [50], the coordinated upregulation of these hydrolases suggests enhanced cell wall turnover during the transition from filamentous to pelletized morphology. Given the role of lipid metabolism in membrane organization and stress tolerance [53], these transcriptional shifts align with the physiological demands of a compact, aggregated growth form, rather than indicating a specific metabolic reprogramming event. Morphologically, pelletized *C. militaris* exhibited restricted hyphal tip extension and increased hyphal aggregation—phenotypes that aligned with active cell wall restructuring rather than unidirectional hyphal elongation. The concurrent increase in chitinase activity supported the interpretation that active cell wall turnover was associated with pellet formation and stabilization, facilitating robust hyphal aggregation into uniform pellets. This heightened enzymatic activity indicated active cell wall remodeling during the maintenance of a compact, aggregated growth form, providing functional support for the transcriptional changes observed in cell wall-related genes. These findings suggest a model in which pelletized growth is linked to coordinated cell wall restructuring and stress adaptation, contributing to improved fermentation performance. Several independent observations in this study were consistent with structural adaptation of the cell wall during pelletized growth. These included increased expression of cell wall-associated genes (Crf1, Chit3, and EglC), elevated chitinase activity, and ultrastructural differences between pelletized and dispersed mycelia observed by SEM. These results support the conclusion that pellet formation is accompanied by significant cell wall restructuring. However, since direct measurements of wall polymer composition and mechanical properties were not performed, the current data are best interpreted as evidence that pelletization is associated with cell wall remodeling-related processes. These expression patterns align with the transcriptomic signatures observed in the RNA-seq analysis and further reinforce the association between pelletized morphology and coordinated transcriptional responses related to stress adaptation and cell wall dynamics [54]. Notably, this validation affirmed the internal consistency of the transcriptomic analysis rather than establishing causal regulatory relationships. It provides an independent line of evidence that the observed transcriptional changes reliably reflect the distinct physiological states of pelletized and dispersed *C. militaris* cultures. These correlations confirm the coherence between the biochemical and transcriptomic profiles, indicating that pelletized and dispersed cultures represent distinct, coordinated physiological states. This integrated analysis describes the coordinated responses associated with pelletized growth, emphasizing that pellet formation is accompanied by broad, interconnected adjustments in stress tolerance and cell wall dynamics, rather than establishing direct causal relationships.

While the described strategy is potentially scalable and cost-effective, several practical challenges warrant consideration. First, at a concentration of 1.5% (*v*/*v*) in the seed culture, P80 represents an additional input. However, since P80 is applied only during the seed stage (typically 5–10% of the total fermentation volume), the overall cost remains modest. For a 100 m^3^ industrial fermenter with a 10 m^3^ seed volume, 1.5% P80 amounts to 150 L per batch. At current market prices (~2–5 USD/L, based on commercial supplier data), this adds approximately 300–750 USD per batch—substantially lower than genetic engineering or alternative morphology control methods. Second, the three-step centrifugation wash increased processing time (approximately 30–45 min) and required a sterile wash station. However, this step was performed only on the seed culture (not the main fermentation), and the added cost was offset by the 71.1% increase in EPS yield and a 24 h reduction in the production cycle. For facilities lacking sterile centrifugation capacity, alternative strategies, such as dilution (e.g., 1:10 transfer without washing), could be explored, although residual P80 carryover would need to be quantified. Third, pellet morphology is known to be sensitive to hydrodynamic shear [55]. Our experiments were conducted in shake flasks at 180 rpm. In stirred tank bioreactors, higher shear could fragment pellets or alter their structure. Scale-up studies using impeller designs that minimize shear (e.g., helical or marine impellers) and controlled aeration would be needed to confirm morphological stability at larger scales. Fourth, P80 is generally recognized as safe (GRAS) by the FDA and is widely used in food, pharmaceutical, and cosmetic products. The acceptable daily intake is 25 mg/kg body weight (JECFA) [56]. Any residual P80 in the final product would likely be negligible due to the wash step and subsequent dilution during fermentation (since the seed culture represents approximately 10% of the total fermentation volume, and P80 is not added to the main fermentation). However, regulatory compliance would require validated analytical methods (e.g., HPLC-MS) to confirm that residue levels are below permissible limits [57]. These practical challenges persist, and future pilot-scale studies should directly address them. Additionally, rheological parameters such as broth viscosity, shear stress, or oxygen transfer coefficient were not directly measured in this study. Our interpretation that pelletized morphology improves mass transfer and reduces viscosity was based on indirect evidence (accelerated glucose consumption, enhanced biomass and EPS yields) and established literature linking pelletized growth to improved fermentation rheology [39]. Direct rheological characterization is necessary to quantitatively confirm these benefits. Future studies should include online or offline viscosity measurements, as well as determinations of the volumetric oxygen transfer coefficient (kLa), to establish a direct correlation between pellet morphology and fermentation rheology.

Several limitations of this study should be acknowledged. First, spore concentration was determined by hemocytometer counting of total spores, and spore viability was not assessed. Consequently, all inoculations were standardized based on total spore count rather than viable count. Viability assessment was not attempted because the experimental design was comparative; the same spore stock and identical handling procedures were used for all groups, ensuring that any potential viability differences were equally distributed. Thus, the relative comparisons between control and P80-treated groups remain unaffected. While the same spore stock was used across all experimental groups, ensuring consistent relative comparisons, variations in initial viability could potentially influence absolute morphological and fermentation outcomes. Future studies should assess spore viability through colony-forming unit (CFU) counts or vital staining to ensure inoculum consistency. Second, analytical measurements (e.g., HPLC-MS) were not performed to quantitatively confirm the complete absence of residual P80 following the three-step washing procedure. Although this washing protocol is standard practice [15], and P80 was absent from the fermentation medium, trace carryover cannot be formally excluded. Future studies should include quantitative residual P80 analysis to definitively rule out direct surfactant carryover effects. Third, while the wash-and-transfer design rules out direct P80 effects during fermentation, it does not separate the contribution of pelletized morphology from the persistence of intracellular changes induced during the seed stage (e.g., transcriptional changes that persist after P80 removal). Thus, the enhanced EPS production could theoretically result from improved mass transfer due to the compact pellet structure (physical mechanism), or from P80-triggered metabolic reprogramming that persists after its removal (biological mechanism), or both. To formally distinguish these possibilities, future studies must compare P80-induced pellets with those generated by alternative methods that do not share the same molecular trigger, such as shear force modulation, pH adjustment, or addition of inorganic particles (e.g., talc or alumina). Additionally, direct rheological and mass transfer measurements (broth viscosity, dissolved oxygen, kLa) were not performed in this study. Although accelerated glucose consumption and earlier peak EPS production are consistent with improved mass transfer, these remain indirect. Future studies should include online or offline viscosity and kLa measurements to quantitatively confirm the physical benefits of pellet morphology, independent of any P80-primed cellular state. Non-biological pellet mimics (e.g., inert beads) could also help assess whether the physical structure alone suffices to replicate the fermentation benefits.

This study presents a simple, cost-effective, and efficient morphological engineering strategy to enhance EPS production in the medicinal fungus *C. militaris*. In contrast to complex genetic modifications, the addition of P80 during seed culture is easy to implement and holds significant industrial potential. For the first time, this study systematically linked morphological regulation, antioxidant capacity, and fermentation performance, elucidating their molecular foundations through transcriptomics and biochemical analyses. A key practical implication is that manipulating seed morphology alone can exert a lasting effect on subsequent fermentation outcomes, even in the absence of surfactant during production, making it an attractive option for industrial deployment. Future studies could explore genetic engineering approaches to overexpress key antioxidant genes (e.g., KatA, GST) or cell wall remodeling genes (e.g., Crf1) identified here, validating their roles in morphology and EPS production. This could lead to genetically stable, high-yield strains without the need for exogenous additives. Additionally, combining P80 with other morphological control methods—such as modulating shear forces or adjusting medium ion concentrations—could further optimize pellet structure and fermentation efficiency. Functional perturbations, such as targeted gene knockouts, RNA interference, or chemical inhibition of specific signaling nodes (e.g., MAPKs) or remodeling enzymes (e.g., Crf1, Chit3), will be necessary to determine whether these pathways play a critical or causal role in the observed morphological and physiological phenotypes. Such studies represent an important direction for future research.

## 5. Conclusions

In conclusion, this study demonstrates that seed-stage morphology induction is sufficient to durably enhance fermentation performance in *C. militaris*. Supplementation with 1.5% P80 during seed cultivation reproducibly established a compact pellet morphology that was retained even after surfactant removal, resulting in a 71.1% increase in EPS yield and earlier peak production. Integrated physiological, biochemical, and transcriptomic analyses support the view that pelletized cultures exhibited a coordinated cellular state associated with active cell wall remodeling and physiological features that likely reflect enhanced stress tolerance or reduced baseline stress exposure. Importantly, the production advantage arises from the induced inoculum morphology, rather than continuous chemical stimulation during fermentation. By combining morphology control with the production stage, this work establishes seed-stage morphology induction as a simple, scalable, and non-genetic strategy for improving fungal bioprocesses. The framework presented here offers new insights for the rational design of high-performance filamentous fungal cell factories.

## Data Availability

The original contributions presented in this study are included in the article/Supplementary Material. Further inquiries can be directed to the corresponding authors.

## Supplementary Materials

The following supporting information can be downloaded at: https://www.mdpi.com/article/10.3390/jof12050362/s1. Table S1: The primers used for qRT-PCR in this study; Table S2. Upregulated and downregulated DEGs between pelletized (1.5% P80) and dispersed (0% P80) seed cultures.
